# Spatial immune composition of tumor microenvironment in patients with pancreatic cancer

**DOI:** 10.1007/s00262-023-03573-6

**Published:** 2023-11-08

**Authors:** Eline S. Zwart, Thomas van Ee, Alsya J. Affandi, Lenka N. C. Boyd, Ernesto Rodriguez, Joke M. M. den Haan, Arantza Farina, Nicole C. T. van Grieken, Laura L. Meijer, Yvette van Kooyk, Reina E. Mebius, Geert Kazemier

**Affiliations:** 1 Department of Surgery, Amsterdam UMC, Location Vrije Universiteit Amsterdam, Amsterdam, The Netherlands; 2 Cancer Center Amsterdam, Amsterdam, The Netherlands; 3 Department of Molecular Biology and Immunology, Amsterdam UMC, Location Vrije Universiteit Amsterdam, Amsterdam, The Netherlands; 4 Amsterdam Institute for Infection and Immunity, Cancer Immunology, Amsterdam, The Netherlands; 5 Department of Pathology, Amsterdam UMC, Location University of Amsterdam, Amsterdam, The Netherlands; 6 Department of Pathology, Amsterdam UMC, Location Vrije Universiteit Amsterdam, Amsterdam, The Netherlands

**Keywords:** Pancreatic cancer, Immunology, Tumor microenvironment, Neoadjuvant therapy

## Abstract

This study examined the composition of the immune microenvironment at different sites within resected pancreas specimens from patients with pancreatic ductal adenocarcinoma (PDAC). Therefore, single-cell suspensions were made from fresh tumor and non-tumorous tissue. Fourteen patients were included from whom twelve PDAC and five non-tumorous samples were obtained. These samples were analyzed with a nineteen marker panel on the Aurora spectral flow cytometer. Furthermore, slides from formalin-fixed paraffine PDACs of eight additional patients were stained with eight markers and analyzed by multispectral imaging. These corresponded to central tumor, periphery of the tumor, i.e., invasive front and resected lymph node and were divided into tumor and adjacent tissue. In the single-cell suspension, a decreased ratio between lymphoid and myeloid cells and between M1 and M2 macrophages was observed in the tumor tissue compared to non-tumorous tissue. Furthermore, an increase in CD169 + macrophages in patients undergoing neoadjuvant therapy was found. Using immunofluorescence, more macrophages compared to T cells were observed, as well as a lower ratio of CD8 to M2 macrophage, a higher ratio of CD4–CD8 T cells and a higher ratio of immune-suppressive cells to pro-inflammatory cells in the PDAC area compared to the adjacent non-tumorous tissue. Finally, there were more immune-suppressive cells in the central tumor area compared to the invasive front. In conclusion, we show a gradient in the immune-suppressive environment in PDAC from most suppressive in the central tumor to least suppressive in distant non-tumorous tissue.

**Graphical abstract:**

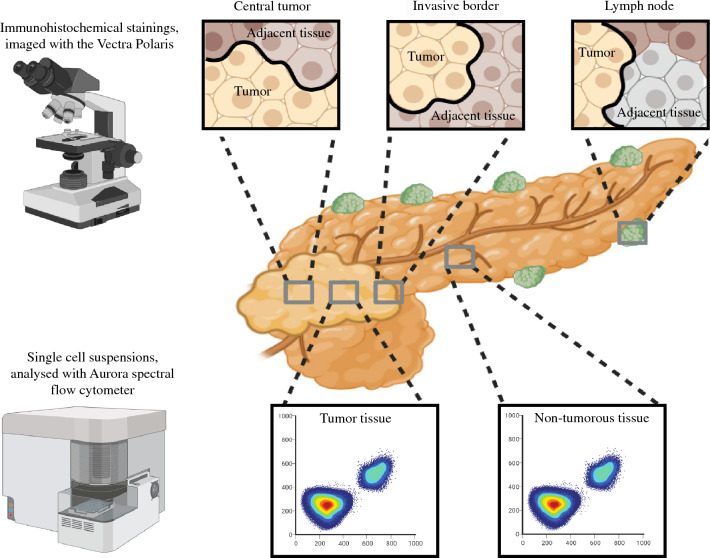

**Supplementary Information:**

The online version contains supplementary material available at 10.1007/s00262-023-03573-6.

## Introduction

Patients with pancreatic ductal adenocarcinoma (PDAC) have a dismal prognosis. Current treatment strategies have improved with the introduction of neoadjuvant chemotherapy, but still the 5-year survival is less than 10% [1–3]. Patients with unresectable tumors have an even worse prognosis with a median survival of 5.8–10.5 months [4]. The key to better treatment strategies appears to be immunotherapy for many carcinoma types [5–9]. However, in patients with pancreatic cancer, these have not granted the desired effect since checkpoint inhibitors, such as programmed death 1 (PD-1), programmed death ligand 1 (PD-L1) and cytotoxic T lymphocyte antigen-4 (CTLA-4), had a very limited effect on survival [10, 11]. Therefore, better understanding of the immune composition of the tumor microenvironment (TME) in PDAC is pivotal to develop effective immunotherapies for pancreatic cancer patients.

It is well known that the TME of PDAC is considered to be immunosuppressive and is rich in desmoplastic stroma. PDAC is considered to be a heterogeneous disease, illustrated by the identification of several molecular subtypes [12–15]. Furthermore, the stroma compartment is as well quite heterogeneous with many subtypes of cancer-associated fibroblasts identified till date [16–18]. However, the immune component of the PDAC TME still needs to be characterized in more detail. Moreover, the gradient of the immune populations has not yet been examined at different sites within one single lesion. This could lead to better understanding of the tumor microenvironment. Spatial differences are relevant as they can influence research results and if present should be taken into account for future research.

We aimed to compare the immune composition of PDAC with non-tumorous pancreatic tissue using single-cell phenotyping focusing on myeloid immune populations. Furthermore, we have also characterized immune populations in different areas of the tumor (central vs. invasive front and lymph node metastases) by multispectral imaging.

## Methods

### Study design

The study design and protocol were approved by the local Medical Ethics Board of the Amsterdam UMC, VU University, Amsterdam, (NL) (2016.59) in accordance with the ethical guidelines of the Declaration of Helsinki. Written informed consent was obtained from all participants before study participation. Fourteen patients undergoing a pancreatic resection between 2018 and 2021 for PDAC at our institute were included for flow cytometry analysis. Fresh tissue was collected at the department of pathology directly after surgery and kept on ice until dissociation. Lesional tissue was macroscopically identified and considered malignant; soft pancreatic tissue was considered non-tumorous tissue. In addition, 4-um slides from formalin-fixed paraffin-embedded (FFPE) PDAC material were obtained from eight additional patients for multispectral imaging. Of each patient, one slide corresponding to the center of the tumor, its periphery (invasive front) and a lymph node metastasis was obtained. Areas containing tumor cells, hereafter called tumor tissue, and adjacent areas without tumor tissue, hereafter called adjacent tissue, were indicated on a hematoxylin and eosin slide by a gastrointestinal pathologist.

### Tissue dissociation

Pancreatic tissue was cut into small pieces and dissociated with RPMI (ThermoFisher Scientific) + DNase 75ug/mL + Collagenase P 0.5 mg/mL + Dispase 80ug/mL + 5% Fetal Bovine Serum (FBS, Biowest) for 45 min at 37 °C. The suspension was filtered through a 100-um strainer, and the tube was rinsed with IMDM + 10%FBS. Cells were centrifuged and washed and frozen in 50% IMDM (ThermoFisher Scientific) + 10%FBS/50% FBS + 22% DMSO using a Mr. Frosty freezing container. Finally, cells were cryopreserved till further analysis in liquid nitrogen.

### Flow cytometry

Cells were resuscitated by rapid thawing in 15 mL pre-warmed RPMI complemented with 20% FBS. After washing and counting, cells were incubated with Human TruStain FcX™ (Biolegend, catalog #422302) and viability dye (LIVE/DEAD™ Fixable Blue Dead Cell; ThermoFisher, Catalog #L34962) prior to cell surface staining with fluorescence-conjugated antibodies in 0.5% BSA/PBS for 20 min at 4 °C. The following surface stain antibodies were used: Anti-CD45–AF700 (Clone 2D1, Biolegend, Catalog #368513), Anti-CD3–BV480 (Clone UCHT-1, BD, Catalog #566105), Anti-CD8-Pacific Orange (Clone 3B5, Life Technologies, Catalog #MHCD0830TR), Anti-CD4–Spark Blue™ 550 (Clone SK3, Biolegend, Catalog #344655), Anti-CD25–APC/Fire™ 810 (Clone M-A251, Biolegend, Catalog #356149), Anti-CD19- BV570 (Clone B159, Biolegend, Catalog #560993), Anti-CD20–AF488 (Clone L26, eBioscience, Catalog #53-0202-82), Anti-CD56–PE-Cy5 (Clone B159, BD, Catalog #561904), Anti-HLA-DR–BV711 (Clone L243, Biolegend, Catalog #307644), Anti-CD14–BV605 (Clone M5E2, Biolegend, Catalog #301834), Anti-CD11c–BV650 (Clone B-ly6, BD, Catalog# 563403), Anti-CD1c–PerCP-ef710 (Clone L161, eBioscience, Catalog #46–0015-42), Anti-CD88–PE-Dazzle594 (Clone S5/1, Biolegend, Catalog #344318), Anti-CD163 – BV421 (Clone GHI/61, Biolegend, Catalog #333612), Anti-CD169–AF647 (Clone HSn 7D2, Novus Biologicals, Catalog #NB600-534AF647), Anti-FcER1a–APC/Fire™ 750 (Clone AER-37 (CRA-1), Biolegend, Catalog #334644). After thorough washes, cells were incubated, and intracellular antibodies Anti-FoxP3–PE (Clone FJK-16s, ThermoFisher, Catalog # 12-5773-80) and Anti-CD68–PE-Cy7 (Clone Y1-82A, Biolegend, Catalog #333815) stained, with eBioscience™ Foxp3/Transcription Factor Staining Buffer Set (ThermoFisher, Catalog # 00-5523-00) in accordance with the manufactures protocol. Finally, cells were fixed with 2% paraformaldehyde for 10 min at 4 °C stored at 4 °C until acquisition. Cells were acquired on a Aurora spectral flow cytometer (Cytek), followed by unmixing in SpectroFlo (Cytek) and pre-processing and manual gating in FlowJo (Treestar, version 10). Only samples with more than 10,000 live cells were included in the analysis. These samples were subsequently analyzed with OMIQ (Omiq, Inc).

### Multispectral imaging

Slides were stained in accordance with the manufactures protocol (Akoya Biosciences, Catalog #NEL821001KT). The following primary antibodies were used: CD3 (Clone Sp7, Abcam, #ab86734), CD8 (Clone C8/144B, Dako Agilent, Catalog #M710301-2), FoxP3 (Clone 236A/E7, EBioscience, Catalog #14-4777-82), CD20 (Clone L26, ThermoFisher, Catalog #14-0202-82), CD68 (Clone PG-M1, ThermoFisher, Catalog #MA5-12407), CD163 (Clone 10D6, ThermoFisher, Catalog # MA5-11458), Ki-67 (Clone MIB-1, Dako, Catalog #M724029-2), CD14 (Clone EPR3653, Abcam, Catalog #ab133335), PanCK (Clone AE1/AE3 + 5D3, Abcam, Catalog #ab86734), in combination with Opal Polaris 480 (Akoya Biosciences, Catalog #FP1500001KT), Opal Polaris 780 (Akoya Biosciences, Catalog #FP1501001KT) and Opal 520, Opal 540, Opal 570, Opal 620, Opal 650 and Opal 690 (Akoya Biosciences, Catalog #NEL821001KT). Slides were imaged using the Vectra Polaris.

### Image analysis

Acquired images were spectrally unmixed using inForm (version 2.6.0, Akoya Biosciences). Image analysis was performed using QuPath (version 0.2.2, Pete Bankhead). Cells were segmented using DAPI and the build-in watershed cell detection. Tumor and adjacent tissue annotations were copied from the annotation on the hematoxylin and eosin (H&E) slide by the dedicated pancreas pathologist. Immune and tumor cell phenotyping was done using Random Trees machine learning per individual marker and ultimately combined to a composite classifier. Immune populations were classified as following: CD3^+^CD20^−^CD14^−^ ‘Total T cells,’ CD3^+^CD8^+^FoxP3^−^ ‘cytotoxic T cells/CD8 T cells,’ CD3^+^CD8^−^FoxP3^+^ ‘regulatory T cells,’ CD3^+^CD8^−^FoxP3^−^ ‘T helper cells/CD4 T cells,’ CD20^+^CD3^−^ ‘total B cells,’ CD20^+^FoxP3^+^ ‘regulatory B cells,’ CD3^−^CD20^−^CD14^+^ ‘myeloid cells,’ CD14^+^CD68^−^CD163^−^ ‘monocytes,’ CD14^+^CD68^+^CD163^−^ ‘M1 macrophages,’ CD14^+^CD68^+^CD163^+^ ‘M2 macrophages,’ and in tumor area CD3^−^CD20^−^CD14^−^PanCK^+^ ‘tumor cells.’

### Statistical analysis

Clinical data were analyzed in SPSS (IBM SPSS Statistics, version 26). For non-skewed continuous variables, the one-way ANOVA test was used and reported with the mean and standard deviation. For skewed continuous variables, the Kruskal–Wallis test was used and reported with the median. Paired samples were analyzed using the related samples Wilcoxon signed rank test. The Pearson's Chi-squared test was used for ordinal and nominal data. Survival data were dichotomized based on an overall survival of < 18 months or ≥ 18 months based on median overall survival in Dutch studies [4, 19]. A *p* value ≤ 0.05 was considered significant.

## Results

### Patient characteristics

For the single-cell suspension analysis, fourteen patients with PDAC were included; their clinicopathological characteristics are shown in Table 1. Of these patients, twelve single-cell suspensions of tumor tissue were available and five single-cells suspensions of non-tumorous tissue. The majority of patients were males, with a mean age of 68.6 years. Furthermore, almost all patients had a tumor located in the head of the pancreas and twelve patients received a biliary drainage prior to the operation. A total of five patients were given neoadjuvant therapy prior to surgery consisting and ten patients received adjuvant therapy. Eight additional patients were included for the multispectral imaging analysis, who were more frequent male, received less biliary drainage prior to surgery and also consisted of mostly tumor located in the head of the pancreas (Table 1).

**Table 1:** Patient characteristics

	Flow cytometry (*n* = 14)	Multispectral imaging (*n* = 8)
Age, mean (SD)	68.6 (9.0)	73.6 (3.8)
Female, *n* (%)	6 (42.9)	1 (12.5)
CA19.9, median (IQR)	409.5 (1429)	218.0 (1765.0)
Bilirubin, median (IQR)	80.0 (134)	148.0 (209.3)
Biliary drainage, *n* (%)	12 (85.7)	3 (37.5)
Neoadjuvant therapy, *n* (%)	5 (35.7)	0 (0.0)
Preoperative infection, *n* (%)	3 (21.4)	0 (0.0)
Location tumor, *n* (%)		
Head	13 (92.9)	6 (75.0)
Tail	1 (7.1)	2 (25.0)
PDAC		
Tumor stage*, *n* (%)		
1A	1 (7.1)	0 (0.0)
1B	1 (7.1)	1 (12.5)
2A	0 (0.0)	0 (0.0)
2B	7 (50)	4 (50.0)
3	5 (35.7)	3 (37.5)
Perineural invasion, *n* (%)	12 (85.7)	6 (75.0)
Angioinvasion, *n* (%)	6 (42.9)	1 (16.7)
Adjuvant therapy, *n* (%)	10 (71.4)	3 (37.5)

*CA19.9* carbohydrate antigen 19.9

*According to the American Joint Committee on Cancer 8th Edition

### Single-cell flow cytometry results

#### Tumor versus non-tumorous tissue

**Fig. 1:**
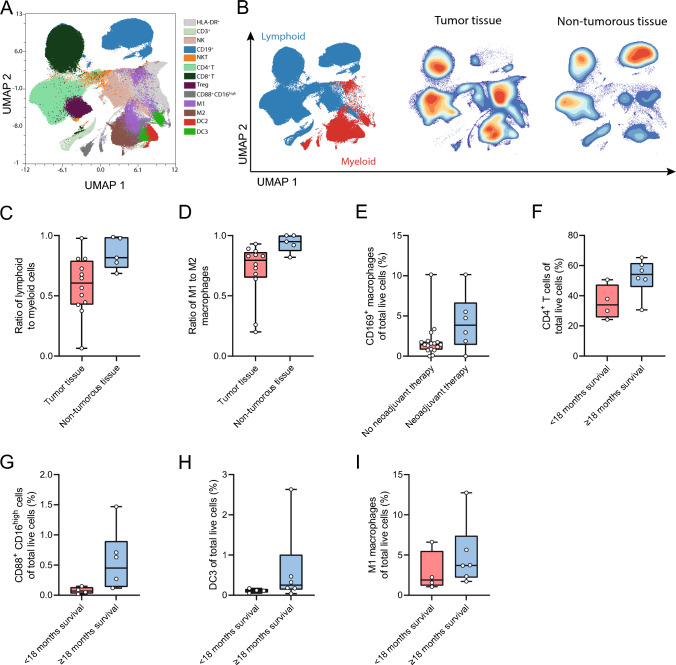
Abundances of immune populations in single-cell suspensions **A** UMAP of included samples, **B** UMAP of lymphoid and myeloid cells, and tumor and non-lesional tissue, **C** ratio between lymphoid and myeloid cells in tumor versus non-lesional tissue, **D** ratio between M1 and M2 macrophages in tumor versus non-lesional tissue, **E** percentage of CD169^+^ macrophages of total cells in patients with PDAC who received neoadjuvant therapy versus those who did not receive neoadjuvant therapy, **F** percentage of CD4 of total cells in tumor tissue of patients with PDAC with < 18-month survival and ≥ months survival , **G** abundances in tumor of PDAC survival CD4 (H) percentage of CD88^+^CD16^high^ of total cells in tumor tissue of patients with PDAC with < 18-month survival and ≥ months survival **I** percentage of DC3 of total cells in tumor tissue of patients with PDAC with < 18-month survival and ≥ months survival , **H** percentage of M1 macrophages of total cells in tumor tissue of patients with PDAC with < 18-month survival and ≥ months survival boxplots: black bar denotes median, box denotes the interquartile range, whiskers indicate the range of values that are outside of the interquartile range. Outliers are defined as > 1.5 times the size of the interquartile range and presented as a*

Using flow cytometry, we compared immune cell composition of tumor versus non-tumorous tissue. For the gating strategy, see Supplementary Fig. 1. These analyses revealed that the abundance of lymphoid cells compared to myeloid cells was significantly lower in the tumor tissue than in the non-tumorous tissue (61.0 vs. 82.0%, *p* = 0.020; Fig. 1A–C). Within the myeloid cells, there was a significant decrease in the abundance of M1 macrophages compared to M2 in the tumor tissue (79.5% vs. 95.0%, *p* = 0.01; Fig. 1D). Thus, within the tumor area there appear to be more lymphoid cells, and the myeloid cells that are present are mostly M2 macrophages.

#### Neoadjuvant therapy

To analyze the effect of neoadjuvant therapy on the immune cell composition, we first compared all samples, including both tumor and non-tumorous tissue, of patients who received neoadjuvant therapy versus those who did not receive neoadjuvant therapy. Receiving neoadjuvant therapy in patients with PDAC significantly increased the percentage of CD169^+^ macrophages compared to patients who did not receive neoadjuvant therapy (3.85% vs. 1.33%, *p* = 0.048; Fig. 1E). Next, we examined the effect of neoadjuvant therapy in tumor samples and non-tumorous tissue samples separately. The significant increase of CD169^+^ macrophages was specifically observed in the tumor tissue from patients with neoadjuvant therapy (4.24% vs. 1.38%, *p* = 0.008). In the non-tumorous tissue, there was no significant difference in any subset of immune cells. Overall, these data suggest that the upregulation of CD169^+^ macrophages is a local effect of neoadjuvant therapy.

#### Survival analysis

Only tumor tissue of patients with PDAC was included in the survival analysis to prevent confounding from spatial differences. In these samples, survival longer than 18 months was associated with higher percentages of CD4 T cells (54.1% vs. 33.95%, *p* = 0.038), HLA-DR^+^CD88^+^CD16^high^ (0.45% vs. 0.07%, *p* = 0.038), HLA-DR^+^CD1c^+^CD88^−^CD14^+^ dendritic cells (DC3) (0.0392% vs 0.008%, *p* = 0.038) and M1 macrophages (3.71% vs. 1.89%, *p* = 0.019) (Fig. 1F-I). Thus, more CD4, DC3 and M1 macrophages within tumor tissue correlate with better survival.

### Multispectral imaging

After showing that there is a more immune-suppressive environment with more M2 macrophages in the tumor compared to non-tumorous tissue, we zoomed into the tumor area itself to unravel intratumor heterogeneity in immune cell composition by comparing central tumor areas to invasive front areas. Furthermore, within these slides we compared tumor areas to adjacent pancreatic tissue, to examine whether the immune-suppressive gradient was also present at these different sites.

#### Tumor tissue versus adjacent tissue

**Fig. 2:**
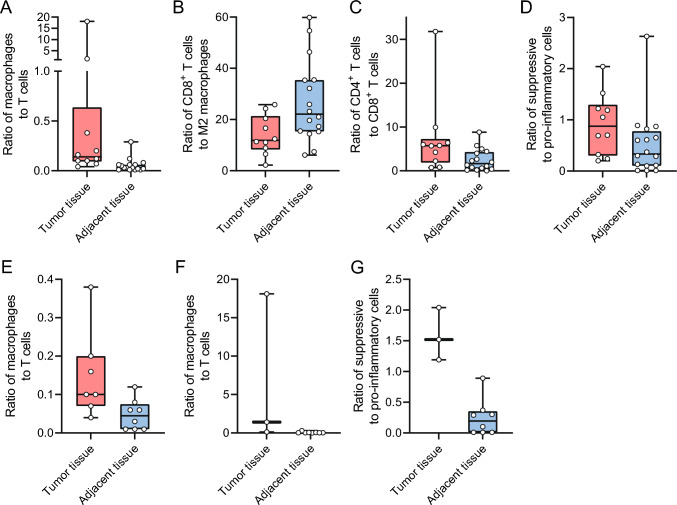
Abundances in tumor tissue versus adjacent tissue **A** percentage of T cells of total cell population, **B** ratio between CD8 T cells and M2 macrophages, **C** ratio between CD4 and CD8 T cells, **D** ratio between suppressive and proinflammatory immune cells, **E** ratio between macrophages and T cells in tumor versus adjacent tissue of central tumor slides, **F** ratio between macrophages and T cells in tumor versus adjacent tissue of invasive border slides, **G** ratio between suppressive and proinflammatory immune cells in tumor versus adjacent tissue of invasive border slides. Boxplots: black bar denotes median, box denotes the interquartile range, whiskers indicate the range of values that are outside of the interquartile range. Outliers are defined as > 1.5 times the size of the interquartile range and presented as a*

First, we examined the differences between areas containing tumor tissue (*n* = 10) and adjacent tissue (*n* = 16) as noted by the pathologist by comparing these from both the invasive front as the central area of the tumor. In tumor tissue compared to adjacent tissue, there were relatively more macrophages compared to T cells (0.14 vs. 0.03, *p* < 0.001; Fig. 2A) and the abundance of CD8 to M2 macrophages was lower (11.89 vs. 22.11, *p* = 0.031 l Fig. 2B). Furthermore, abundance of CD4 cells to CD8 cells is higher in the tumor tissue (5.74 vs. 1.65, *p* = 0.023; Fig. 2C) as well as the ratio of suppressor cells to pro-inflammatory cells (0.88 vs. 0.34, *p* = 0.041; Fig. 2D). Specifically in the center of the tumor, there were relatively more macrophages compared to lymphocytes in the tumor (*n* = 7) compared to the adjacent tissue (*n* = 8) (0.1 vs. 0.045, *p* = 0.021; Fig. 2E). This was also the case when comparing the macrophage to lymphocyte ratio at the invasive front (1.41 vs. 0.03, *p* = 0.024; Fig. 2F). Furthermore, the ratio of immune-suppressive cells to pro-inflammatory cells was still higher in the tumor tissue (*n* = 3) compared to adjacent normal tissue (*n* = 8) (1.52 vs. 0.195, *p* = 0.012; Fig. 2G).

**Fig. 3:**
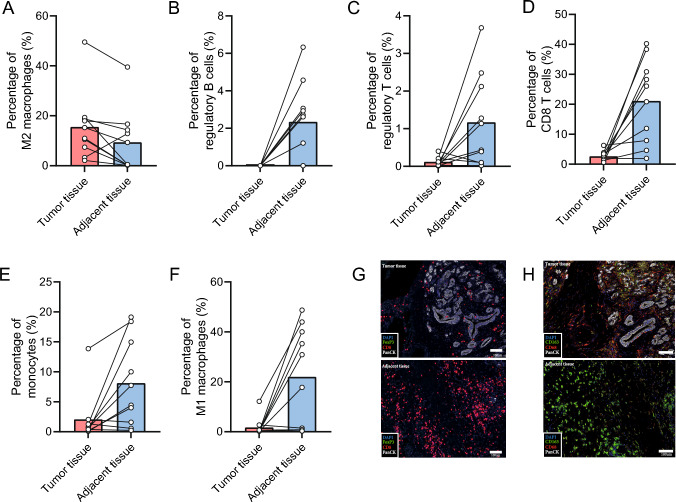
Paired mean abundances of tumor tissue versus adjacent tissue **A** paired mean of M2 macrophages of total cells, **B** paired mean of regulatory B cells of total cells, **C** paired mean of regulatory T cells of total cells, **D** paired mean of CD8 T cells of total cells, **E** paired mean of monocytes of total cells, **F** paired mean of M1 macrophages of total cells, **G** representative images of lymphoid markers of matches tissue, **H** representative images of myeloid markers of matches tissue

Subsequently, we performed a paired analysis with both tumor and adjacent tissue. In the paired samples (*n* = 10), there were a higher percentage M2 macrophages in the tumor tissue (15.5 vs. 9,4, *p* = 0.03), and a lower percentage of regulatory B cells (0.0 vs. 2.3, *p* = 0,006) and T cells (0.1 vs. 1.2, *p* = 0.026), cytotoxic T cells (2.7 vs. 21.1, *p* = 0.003), monocytes (2.1 vs. 8.1, *p* = 0.015) and M1 cells (1.7 vs. 22.0, *p* = 0.009) (Fig. 3A–F) in the tumor tissue. Thus, closer to the tumor cells, there are relative more macrophages, more M2 macrophages and relative more CD4 T cells and less B lymphocytes and CD8 T cells, suggesting an immune-suppressive environment. These results are comparable to the single-cell immunophenotyping experiments.

#### Invasive front versus center of the tumor

**Fig. 4:**
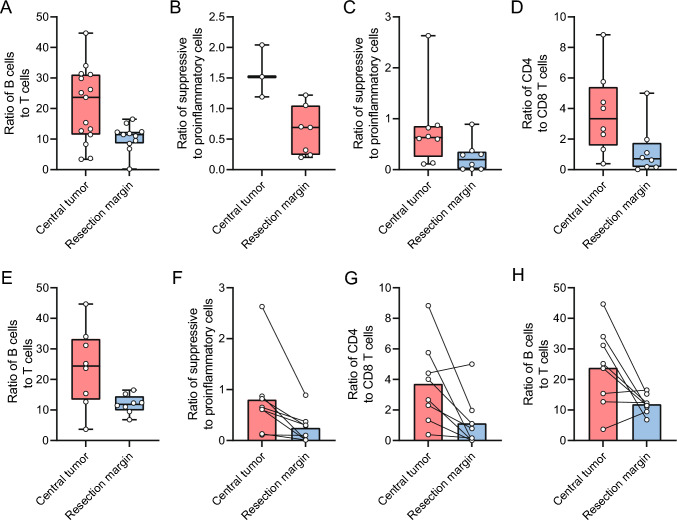
Abundances in central tumor slide versus invasive border slide **A** ratio between B and T cells, (**B**) ratio between suppressive versus proinflammatory immune cells in tumor tissue, **C** ratio between suppressive versus proinflammatory immune cells in adjacent tissue, **D** ratio between CD4 and CD8 T cells in adjacent tissue, **E** ratio between B and T cells in adjacent tissue, **F** paired mean of ratio between suppressive versus proinflammatory immune cells in adjacent tissue, **G** paired mean of ratio between CD4 and CD8 T cells in adjacent tissue, **H** paired mean of ratio between B and T cells in adjacent tissue. Boxplots: black bar denotes median, box denotes the interquartile range, whiskers indicate the range of values that are outside of the interquartile range. Outliers are defined as > 1.5 times the size of the interquartile range and presented as a*

To examine the gradient of the immune-suppressive microenvironment from the center to the border, we compared slides from the center of the tumor (*n* = 15) and the invasive front (*n* = 11). In the central tumor, there was a significant higher ratio of B cells compared to T cells (23.6 vs. 11.5, *p* = 0.023; Fig. 4A).

When comparing only the tumor tissue of the central tumor area (*n* = 7) and the invasive front (*n* = 3), there were relatively more immune-suppressive cells in the central tumor area (1.52 vs. 0.69, *p* = 0.033; Fig. 4B). Remarkably, there was a higher ratio of immune-suppressive cells in the adjacent tissue in the center of the tumor (*n* = 8) compared to the adjacent tissue at the invasive front (*n* = 8) (0.63 vs. 0.195, *p* = 0.05; Fig. 4C). In addition, there was a higher ratio of CD4 T cells compared to CD8 T cells (3.33 vs. 0.71, *p* = 0.028; Fig. 4D) and a higher ratio of B cells compared to T cells (24.4 vs. 11.86, *p* = 0.028; Fig. 4E) in the non-tumorous tissue surrounding the central part of the tumor compared to the non-tumorous tissue surrounding the invasive front.

In the paired adjacent tissue samples (*n* = 8) there was also a higher ratio of immune-suppressive cells to pro-inflammatory cells in the central tumor slide compared to the invasive front slide (0.8 vs. 0.24, *p* = 0.025; Fig. 4F). Furthermore, there was a higher ratio of CD4 T cells to CD8 T cells (3.71 vs. 1.12, *p* = 0.026; Fig. 4G) and B cells to T cells ratio (23.80 vs. 11.89, *p* = 0.036; Fig. 4H). These results demonstrated that not only there is a more immune-suppressive environment in tumor tissue compared to adjacent tissue, but that this also extends over larger distances from the center of the tumor in comparison with the invasive front.

#### Lymph nodes metastases

**Fig. 5:**
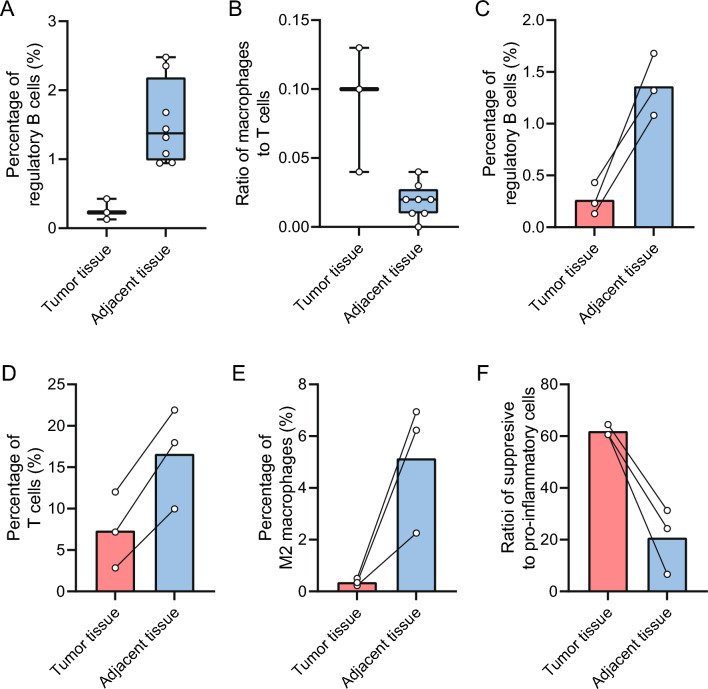
Abundances in lymph node tumor tissue versus adjacent tissue **A** percentage of regulatory B cells of total cells, **B** ratio between macrophages and T cells, **C** paired mean of regulatory B cells of total cells, (D) paired mean of regulatory T cells of total cells, **E** paired mean of M2 macrophages of total cells, **F** paired mean of ratio between suppressive versus proinflammatory immune cells. Boxplots: black bar denotes median, box denotes the interquartile range, whiskers indicate the range of values that are outside of the interquartile range. Outliers are defined as > 1.5 times the size of the interquartile range and presented as a*

To examine the effect of lymph node metastases on the lymph node microenvironment, we compared tumor areas to adjacent lymph node areas as denoted by the pathologist. There was a lower percentage of regulatory B cells in the tissue containing tumor cells (*n* = 3) compared to the adjacent lymphoid tissue (*n* = 8) (0.23 vs. 1.38 *p* = 0.012; Fig. 5A). In addition, there was a significant higher ratio of macrophages to T cells in the tumor tissue (0.1 vs. 0.02, *p* = 0.012; Fig. 5B) and a higher ratio of myeloid cells versus lymphoid cells (0.02 vs. 0.005, *p* = 0.012). This is comparable to the distribution in the pancreatic tissue. Contrary, within paired samples of normal and tumor within the same specimen (*n* = 3), there was a lower percentage of regulatory B cells (0.23 vs. 1.35, *p* = 0.025), T cells (7.34 vs. 16.60, *p* = 0.014) and M2 macrophages (0.35 vs. 5.14, *p* = 0.004) in the tumor tissue compared to the adjacent normal issue (Fig. 5C–E). However, in the area containing tumor cells, there was a higher ratio of immune-suppressive cells versus pro-inflammatory cells (61.94 vs. 20.76, *p* = 0.24; Fig. 5F), suggesting that even though the individual abundances are higher in the adjacent tissue, the ratio compared to proinflammatory cells is still higher in the tumor tissue. Overall, this shows that the immune-suppressive effect appears to extend to the lymph node areas containing tumor cells.

## Discussion

This study is the first to show that there is a gradient in the immune-suppressive microenvironment in patients with pancreatic cancer, where the most immune-suppressive environment is found in the central tumor area, and becomes less immune-suppressive further away toward the invasive front of the tumor and non-tumorous tissue. The immune-suppressive profile consists of more macrophages than T cells specifically more M2 macrophages compared to CD8 cytotoxic T cells. In the macrophage subgroup, there are more M2 macrophages within the central tumor areas. Furthermore, by comparing the tumor tissue and surrounding non-tumorous tissue in the single-cell phenotyping analysis, we were the first to show that this difference in immune profile extends over a larger distance. Additionally, we are the first study to show CD169^+^ macrophages in PDAC tissue and show an increase in patients receiving neoadjuvant therapy.

The increase of M2 macrophages and regulatory T cells in the tumor tissue compared to the normal tissue contributes to the pro-tumorigenic micro-environment. These cells are intertwined in their presence and function. The M2 macrophages produce, among others, IL-10, TGF-*β*, IL-6, PGE, CCL2, CCL17 and CCL20 [20, 21]. These cytokines inhibit pro-inflammatory cells such as cytotoxic CD8 + T cells and DCs, but also push naïve T cells to differentiate into regulatory T cells [22, 23]. These regulatory T cells also produce IL-10 and TGF-*β*, which in turn stimulates the polarization of macrophages into M2 macrophages. These macrophages are important for the tumorigenesis, development of metastasis and contribute to chemoresistance. The increase of M2 macrophages and regulatory T cells has been shown in before, but the gradient between multiple locations that we show here for the first time, adds a new layer of nuance. Väyrynen et al. did show that a closer proximity of M2 macrophages to the tumor cells correlates with worse disease-free survival [24]. Furthermore, Papalampros et al. compared central tumor with invasive front in the same slide and showed more CD4^+^ T helper cells compared to CD8^+^ cytotoxic T cells in the central tumor area and no difference between T regulatory cells between both locations [25]. In our central tumor slide, we did not see a significant difference between the tumor area and the adjacent non-tumorous tissue area. However, when we combined the central tumor slide area and invasive border slide, we did see relatively more M2 compared to CD8^+^ T cells and more CD4^+^ compared to CD8^+^ T cells in the tumor tissue compared to the adjacent tissue. Masugi et al. also showed less CD8^+^ in the central tumor area compared to the border [26]. However, they did not include macrophage markers in their panel; therefore, no ratios have been provided.

We show the presence CD169^+^ macrophages in a single-cell suspension of PDAC tissue. CD169 mediates the adhesion and binding between cells to facilitate cellular communication through sialylated ligands, and it also serves as a receptor for sialylated pathogens. They have been described intra-tumorally in hepatocellular carcinoma and gastric cancer and suggest a correlation with good survival and favorable TNM stage [27]. In this explorative study, we did not find a correlation with survival and the subgroups were too small to correlate CD169^+^ macrophages with TNM stage. Furthermore, CD14^+^CD169^+^ cells have been detected as circulating antigen-presenting cells in peripheral blood mononuclear cells (PBMCs) of patients with PDAC [28]. We show an increase of these cells in pancreatic cancer tissue of patients receiving neoadjuvant therapy. As neoadjuvant therapy leads to tumor apoptosis and these macrophages have been shown to capture tumor-derived material, it could be hypothesized that the increase in CD169^+^ macrophages has a favorable role in response to neoadjuvant therapy. These CD169^+^ macrophages are associated with antigen-specific T cell activation, and there is a positive correlation between the density of CD169^+^ macrophages and CD8^+^ T cell infiltration in tumor and tumor draining lymph nodes [27, 29, 30]. As this study only contained four patients who received neoadjuvant therapy, future research is needed to examine the correlation between the abundance of CD169^+^ macrophages and effectiveness of neoadjuvant therapy. However, possible stimulation of CD169^+^ macrophages might be an interesting target for immunotherapy as the uptake of tumor cell material that is released after cell death caused by the neoadjuvant therapy by CD169^+^ macrophages has been shown to further stimulate anti-tumor immunity [31].

We found a positive correlation between survival and an increase in DC3s and M1 macrophages. Both cells provide a pro-inflammatory immune profile which could contribute to better survival. In many other malignancies, M1 macrophages are associated with better survival [32–35]. DC3 is a DC subset that shares expression of CD1c and Fc*ε*RI with DC2, but it can be distinguished by CD14^+^ and CD163^+^ expression, and it has been described to have a pro-inflammatory phenotype [36]. Furthermore, DC3s have been described to activate tumor antigen-specific T cells [36]. They can also produce high IL-12 upon IFN-y release by neighboring T or NK cells, which then stimulates CD8^+^ T cells [37, 38]. In addition, gene expression of DC3 is also associated with better survival in lung cancer [39]. Furthermore, we also found that an increase of CD4 T cells was correlated with better survival in this small patient cohort. Even though this might seem contra-intuitive due to the immune-suppressive effect of regulatory T cells, an increase in intratumoral CD4^+^ T cells has been described to associate with better survival in patients with PDAC [40–42]. In these studies, higher CD4^+^ T cells were associated with better survival, but FoxP3^+^ cells were not, suggesting a different role for the intratumoral CD4^+^ T cells. This has also been shown by Carstens et al., who show that high levels of T cell infiltration, cytotoxic T cells and CD4^+^ T effector cells are correlated with a better prognosis, but no correlation with regulatory T cells was found [43]. It has been suggested that these CD4^+^ T cells play a pivotal role in the induction and maintenance of CD8^+^ cytotoxic T cells [44].

The immune composition of especially T cells can also be influenced by the presence of PD-1/PD-L1. The PD-1/PD-L1 axis plays a pivotal role in the inhibition of effector T cells and the development of regulatory T cells [45]. If PD-L1 is present, it is correlated with a higher abundance of CD3, CD4, CD8, FoxP3-positive cells [46]. In PDAC, PD-1 is expressed on the majority of regulatory and CD4 T cells within the tumor-infiltrating lymphocytes, but there presence on CD8 T cells in contradictory as both high abundance and very low abundance has been described [47, 48]. Of note, the CD8^+^PD1^+^ T cells appear to be only present in tertiary lymphoid structures in tumor stroma and peripheral tumor and not in the pancreatic tissue itself [47]. High PD-L1 expression is associated with worse survival, possible due to immune-suppressive effect [46].

As we show that the immune composition differs between different locations within one pancreatic resection specimen, it is pivotal to specify the location of the examined sample in future research instead of simply mentioning tumor vs normal tissue, as this gradient could affect the results, hampering comparisons of the immune microenvironment. Furthermore, as the immune composition differs within one tumor, this might be important for the development of immunotherapy as it might induce a different effect at different sites of the tumor based on the presence of the targeted immune cell subset. In addition, many studies have shown a high intratumoral heterogeneity in the PDAC immune microenvironment, even in small areas within one single slide [26, 42]. To correct for this heterogeneity, we not only examined the different sites within one resected specimen, but also performed whole slide analysis rather than TMA analysis. This has ensured that no selection bias within the samples could affect the acquired results.

One of the limitations of this study was the number of cells in each sample. Unfortunately, the size of the available tissue was small, leading to limited cell numbers. Not only might this have led to missing rare immune populations, but samples had to be excluded from the final analysis due to overall low cell counts. As a cutoff of ≥ 10,000 viable cells was used in the single-cell suspension, the possibility of missing rare immune populations is minimized as much as possible. In addition, the sample size of this explorative study is small, especially in the neoadjuvant treated group. This is partly due to the exclusion of samples that had a low viable cell count. Furthermore, it appears that not all macrophages survive the freeze and thawing process, leading to perhaps skewed number of the other cell populations. Finally, due to the high autofluorescence nature of the single cells derived from tumor tissue, such as myeloid cells, tumor and stromal cells, improved spectral unmixing methods can be implemented in future studies [49].

In conclusion, it appears to be a gradient of immune-suppressive environment in pancreatic cancer, from the most immune suppressive in the central tumor area, to less immune suppressive in the invasive front and distant non-tumorous tissue. This finding not only confirms our hypothesis, but adds a layer of complexity that should be dealt with in future translational research. Finally, this is the first study to show an increase of CD169^+^ macrophages in patients receiving neoadjuvant therapy, which might be an interesting biomarker for the efficacy of neoadjuvant therapy.

### Supplementary Information

Below is the link to the electronic supplementary material.

Supplementary file1 (PDF 688 KB)

## Data Availability

The data used during the current study are available from the corresponding author on reasonable request.
