# Supplementary figures and images for: Spatial immune composition of tumor microenvironment in patients with pancreatic cancer
Source: Cancer Immunol Immunother. 2023 Nov 8;72(12):4385–97. doi: 10.1007/s00262-023-03573-6 (PMC10700423; doi:10.1007/s00262-023-03573-6)

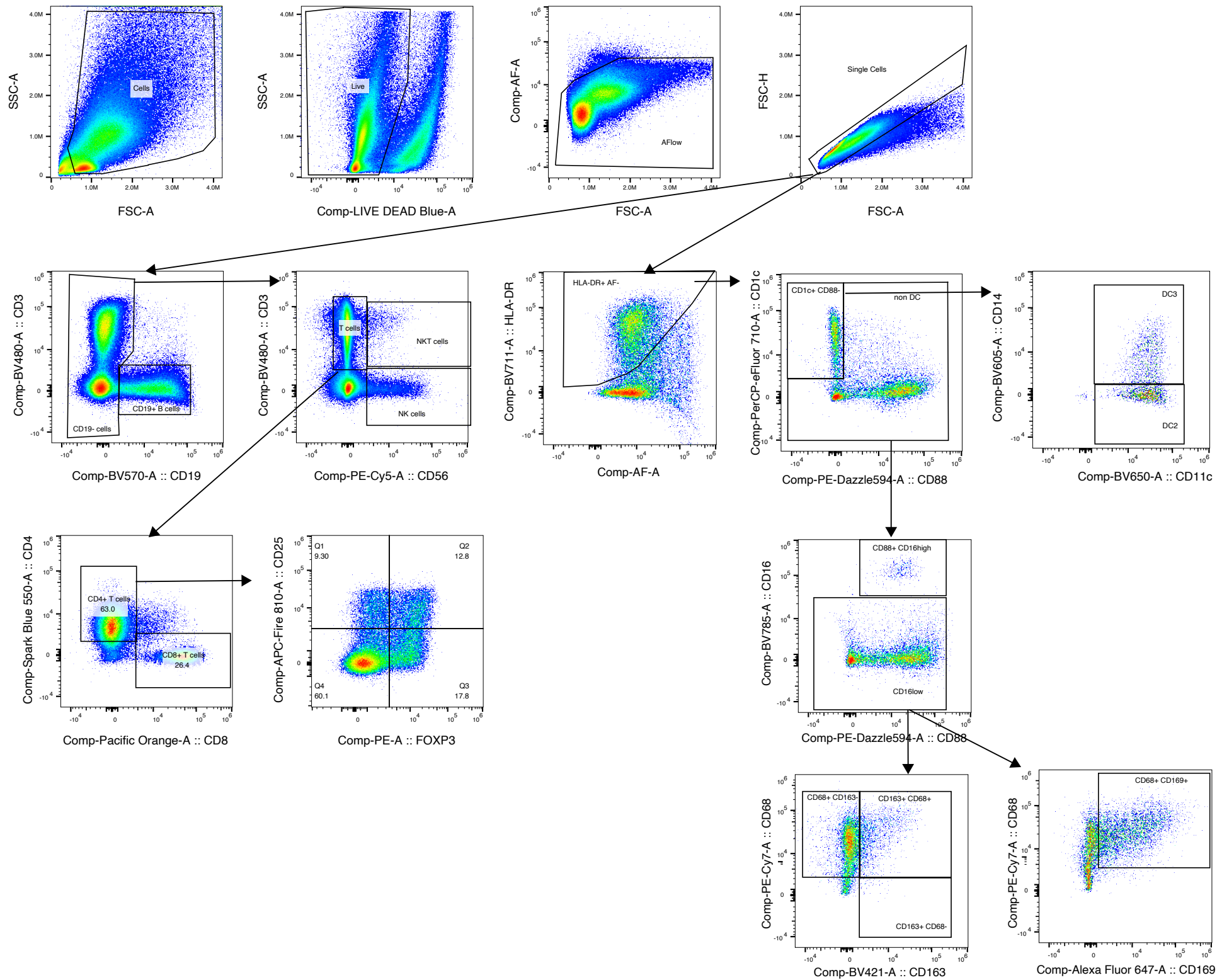

Supplement: Supplementary file 1 — Supplementary file1 (PDF 688 KB) [file 262_2023_3573_MOESM1_ESM.pdf]
